# Beyond the needle: How nonsurgical management transforms foot and ankle ability in male footballers with posterior ankle impingement- a longitudinal study

**DOI:** 10.1016/j.heliyon.2024.e40484

**Published:** 2024-11-16

**Authors:** Jyoti Gupta, Moattar Raza Rizvi, Ankita Sharma, Waqas Sami, Noof Fahad A Al-Kuwari, Fatma Hegazy, Shahnaz Hasan

**Affiliations:** a Department of Physiotherapy, School of Allied Health Sciences, Manav Rachna International Institute and Studies (MRIIRS), Faridabad, 121001, India; b Department of Pre-Clinical Affairs, College of Nursing, QU Health Sector, Qatar University, Doha, P.O. Box 2713, Qatar; c Department of Physiotherapy, University of Sharjah, Sharjah Emirate, United Arab Emirates; d Department of Physiotherapy and Health Rehabilitation, College of Applied Medical Sciences, Majmaah University, Al-Majmaah, Kingdom of Saudi Arabia; e Department of Physiotherapy, Amity Institute of Allied Health Sciences, Amity University, Noida, 201303, India; f College of Healthcare Professions, Dehradun Institute of Technology (D.I.T) University, Mussoorie, Diversion Road, Makka Wala, Uttarakhand, 248009, India

**Keywords:** Posterior ankle impingement, Foot and ankle ability measure, Football players, Corticosteroids, Nonsurgical management

## Abstract

**Purpose:**

Posterior ankle Impingement (PAI) is a condition commonly affecting athletes and individuals engaged in activities involving repetitive ankle plantar flexion. This study aimed to evaluate the impact of nonsurgical management, excluding corticosteroid injections, on the functional outcomes of football players diagnosed with PAI.

**Materials and methods:**

Twenty male football Players between age group of 18–30 years, clinically diagnosed of PAI, were included in this longitudinal study. Nonsurgical management was implemented over 8 weeks, including exercises. Players were assessed for pain, tenderness, range of motion(ROM), Foot and Ankle Ability Measure (FAAM), vertical and broad jump height at baseline, after 4 weeks and 8 weeks.

**Results:**

Significant improvements were observed in pain levels (p < 0.001), tenderness (p < 0.001), ROM (p < 0.001), and functional abilities measured by FAAM ADL (p < 0.001) and Sports subscales (p < 0.001) after 8 weeks of nonsurgical management. Horizontal jump performance significantly improved (p = 0.02), while vertical jump did not (p = 0.15) after 8 weeks. Tenderness and ROM showed significant changes after 4 and 8 weeks. The FAAM% score significantly increased (p < 0.001) after 8 weeks, reflecting enhanced overall functional ability.

**Conclusions:**

Nonsurgical management of 8 weeks is an effective approach for ameliorating symptoms and enhancing functionality in football players with PAI as compared to 4 weeks.

## 1 Introduction

Football is a high-intensity, contact sport that demands a lot from its players. FIFA reports approximately 265 million players worldwide as of 2007 [1]. The sport requires sustained aerobic activity and diverse neuromuscular skills like jumping, kicking, turning, and sprinting, placing significant strain on the body and increasing injury risk. Research indicates that football accounts for one-fourth to one-half of all sports-related injuries in Europe [2], with some studies noting as high as 70 % in specific populations, though it can be as low as 20 % or 30 % [3] given its high-risk, injury prevention is essential for player safety and performance.

Ankle injuries are common in football, with sprains being the most prevalent. Among football players, ankle injuries account for 11 %–25 % of acute injuries [4]. Another common type is posterior ankle impingement (PAI) often seen in athletes performing repetitive plantarflexion movements, such as running, jumping, and kicking. PAI is characterized by deep pain at the back of ankle typically caused by compression of soft tissue structures between the talus, tibia and posterior aspect of calcaneum during plantar flexion. This condition can become chronic, especially with repeated strain from football activities. The prevalence of PAI in football players varies widely, from 5 % to 60 %, influenced by the study population and the diagnostic criteria used [5]. While the primary mechanism of pain in PAI is related to plantar flexion, forced dorsiflexion can also be associated with pain in some patients where the structures along the anterior margin of the tibiotalar joint are impinged. Dorsiflexion is not directly related to the posterior impingement posterior soft tissue structures or the pain experienced in PAI. However it can be affected by the condition and may be limited due to the associated inflammation, scarring, or hypermobility of the soft tissue structures [6].

To compensate for the plantarflexion loss, athletes with PAI may inwardly rotate their feet, risking ankle sprains, calf sprains, contractures, and plantar foot pain [7]. PAI may also result from an os trigonum or Stieda's process at the back of the ankle [8], and is associated with other issues like fractures in the lateral tubercle of the posterior talar process, compression of the posterior soft tissues, tension on the posterior talofibular ligament (PTFL) and posterior capsule, as well as inflammation of the flexor hallucis longus (FHL) tendon or tenosynovitis. Additional related conditions include ankle osteochondritis, subtalar joint disease, further fractures, inversion trauma/sprain, and posterior tibiotalar compression syndrome [6,8,9]. Football players often exhibit posterior ankle pain that worsens with plantarflexion, tenderness on the ankle’s posterior and tenosynovitis of the flexor hallucis longus (FHL) [8]. Radiographic imaging, such as X-ray, magnetic resonance imaging (MRI), and computed tomography (CT) scans, are essential for diagnosing PAI. X-rays can identify bony anomalies such as osteophytes or an os trigonum due to over-projection MRI and CT scans provide detailed views of soft tissue like the posterior capsule, synovium, and FHL tendon, crucial for identifying impingement sources [10].

The heel thrust test, used to evaluate PAI, places the ankle in maximal plantar flexion with the patient prone, applying overpressure with an inversion/eversion bias for less irritable patients. While widely used, the test ‘specificity and sensitivity are not documented. The Foot and Ankle Ability Measure (FAAM) is a validated tool used to assess the functional abilities in individuals with foot and ankle conditions, including PAI. Its sensitivity to changes in daily and sports-related activities makes it an ideal measure for evaluating the effectiveness of nonsurgical treatments in football players [11,12].

Studies have shown that nonsurgical management, including physical therapy, can be effective in treating high ankle injuries and improving functional outcomes in football players [13]. Nonsurgical management of PAI typically combines rest, ice, compression, and elevation (REST), along with physical therapy and nonsteroidal anti-inflammatory drugs (NSAIDs) for pain and inflammation management [9]. Although corticosteroid injections may provide short-term relief by reducing inflammation and pain, they do not address the underlying cause of the PAI and could potentially lead to further damage if used excessively. Therefore, these injections should be administered judiciously, complementing other nonsurgical measures such as physical therapy and rest, to manage symptoms effectively and target the root cause of the PAI. Physical therapy may enhance range of motion, muscle strength, proprioception and balance. Techniques such as ultrasound and electrical stimulation are also employed to aid healing and alleviate pain [14,15].

Despite the recognized efficacy of these nonsurgical strategies, there remains a significant gap in the literature concerning their specific impacts on functional abilities, particularly as measured by validated tools like the FAAM. Current studies often lack comprehensive evaluations that can provide real-world insights into treatment effectiveness. Addressing this gap is crucial, as it aids clinicians in selecting the most appropriate management strategies and enhances our understanding of how these treatments can be tailored to meet the specific needs of football players suffering from PAI. Consequently, the primary objective of this study was to evaluate the efficacy of nonsurgical management strategies for male football players diagnosed with PAI, specifically by assessing changes in their functional abilities through the FAAM.

## 2 Methodology

### 2.1 Participants

**Fig. 1:**
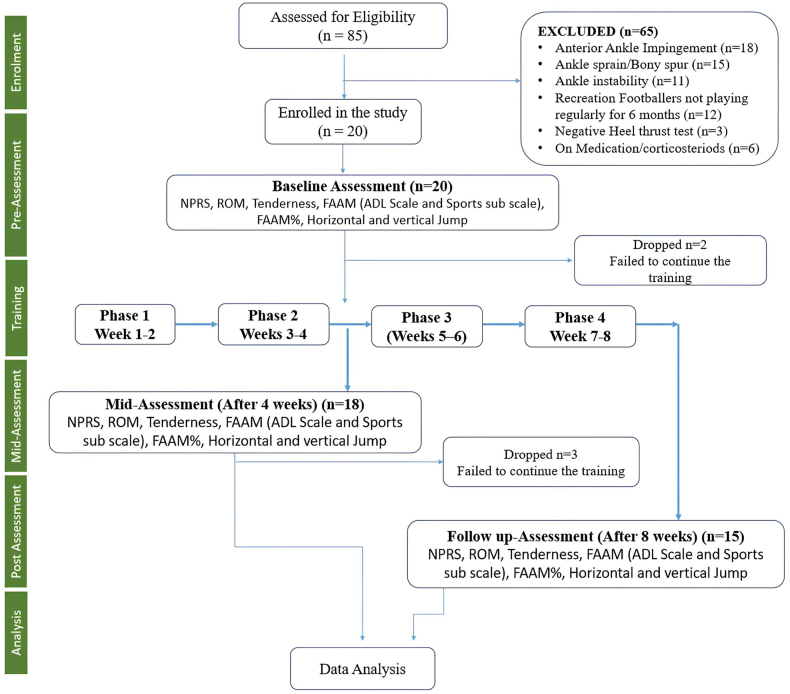
Study design.

The study was conducted from February 1, 2022 to August 30, 2022. To achieve the desired sample size of 20 players, a total of 85 male football players visiting Outpatient Department at Manav Rachna Institute of Research & Studies were initially screened for confirmed diagnosis of posterior ankle impingement (PAI). These players were screened based on the reported pain (mild to moderate), discomfort in the posterior ankle region (Fig. 1). The study recruited players with symptomatic but not severely debilitating PAI. Many of these athletes were actively managing their discomfort through various means while continuing their football pursuits.

**Fig. 2:**
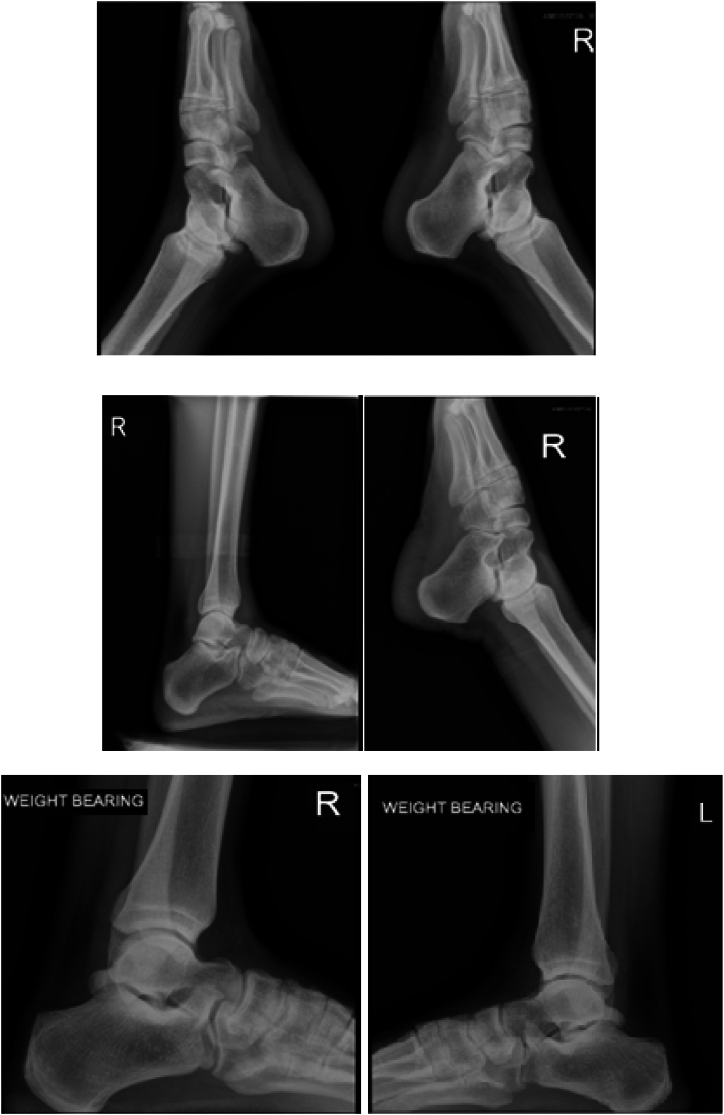
Series of X-ray images demonstrating various views of the ankle joints. Top row: Anteroposterior views of both ankles, showing normal bony alignment without signs of fracture or significant degenerative changes. Middle row: Lateral views of the right ankle in different flexion positions, highlighting the clear spaces and bone densities. Bottom row: Weight-bearing views of both the right (R) and left (L) ankles, useful for assessing alignment under physiological load conditions. These images were accessed for inclusion to rule out other bony abnormalities or other related pathologies.

This was longitudinal study where the outcome variables were measured at baseline, after 4 weeks and 8 weeks. The diagnosis of PAI typically involved a combination of patient’s history, physical examination, and imaging (X-ray) (Fig. 2). During the patient history, the patient's symptoms, such as pain in the posterior ankle during repetitive plantar flexion or jerky activities that involve ankle plantar flexion were observed. History of any previous ankle injuries, surgeries, or other medical conditions was examined. Physical examination typically involved a series of maneuvers to assess ankle range of motion, stability, and tenderness.

All participants in this study underwent detailed physical examination to confirm the diagnosis of posterior ankle impingement focusing on specific clinical indicators, such as localized tenderness behind the peroneal tendons and the reproduction of pain through forced passive plantar flexion of the ankle. The heel thrust test was also employed as part of our diagnostic toolkit to assess the integrity and functionality of the posterior ankle structures by provoking pain. This test was particularly useful in detecting significant pain, weakness, or difficulty in plantar flexion against resistance, which could indicate broader issues related to the posterior tibial muscle group often associated with PAI. Additionally, all participants received ankle radiographs, including a lateral view in full plantar flexion to identify the presence of os trigonum or other anatomical features contributing to PAI. More advanced imaging studies are not usually required for diagnosis [16]. The use of lidocaine injections was limited to cases where diagnostic clarity was needed and was not uniformly applied to all participants.

### 2.2 Sample size calculation

A priori power analysis was conducted using G∗Power software to determine the required sample size for a repeated-measures ANOVA within interaction at three different time points (0 weeks, 4 weeks, and 8 weeks). The study aims to investigate the effect of a within-subjects factor on the outcome variable over time. A desired power level of 0.80 and alpha level of 0.05 were chosen based on the research question and the expected effect size. The assumptions for the power analysis included an effect size of 0.25, a correlation of 0.5 between repeated measures, and the F-test as the statistical test. The power analysis indicated that a total sample size of 20 participants would be required to achieve the desired power level at each time point.

### 2.3 Inclusion criteria

Male football players between age group of 18–30 years clinically diagnosed of PAI during clinical examination with symptomatic presentation of mild to moderate pain (<6 on NPRS scale) on posterolateral aspect of ankle, stiffness and painful plantarflexion range of motion. The presence of os trigonum as seen on x-ray was taken into consideration for the confirmation of posterior ankle impingement. The study included only athletes who were actively participating in football activities, such as training sessions and matches, to ensure that the nonsurgical management strategies are being applied in a relevant context. The heel thrust test was used to evaluate the integrity of the tibial nerve and the posterior tibial muscle in the lower leg for including in the present study. A positive test result was determined if the player exhibited an inability to plantarflex the foot against resistance, substantial weakness, or experienced significant pain in the posterior aspect of the ankle during the test.

### 2.4 Exclusion criteria

Players with diagnosis not specific to posterior ankle impingement syndrome, e.g. anterior ankle impingement or achilles tendon pathology, previous ankle surgery, significant ankle instability, systemic conditions such as diabetes or peripheral neuropathy, and those on certain medications that may affect nerve function or muscle strength, such as neuromuscular blocking agents or corticosteroids were excluded. Ankle instability is often associated with ligamentous injuries, such as sprains however, the objective measurement of the mechanical component of ankle instability is still a matter of scientific debate.

## 3 Testing evaluations

### 3.1 Range of motion (ROM)

To measure the plantar flexion ROM, the players were instructed to assume a prone position on a flat surface with their legs extended straight out behind them [17]. The leg was to be supported by holding the heel with one hand and the forefoot with the other hand. Slowly plantarflex the player's foot towards the shin and measured the angle at which the player experienced pain or discomfort using a goniometer. The fulcrum of the goniometer was centered over the lateral malleolus while the stationary arm was aligned with the long axis of the fibula and the movement arm was parallel to the fifth metatarsal. The angle of plantar flexion at which the player experiences discomfort was recorded as their plantar-flexion ROM [18].

### 3.2 Numeric pain rating scale (NPRS)

NPRS was used to assess pain intensity in football players. The player was asked to rate their pain on a scale of 0–10, with 0 indicating no pain and 10 indicating the worst pain imaginable. The players were provided with a visual aid, such as a chart, to help them understand the scale [19].

### 3.3 Tenderness

Systematic palpation was performed using the fingers to search for areas of tenderness or discomfort and the player was asked to report any pain or discomfort experienced during the assessment [20]. The tenderness was recorded on a scale of 0–4, where 0 indicates no tenderness and grade 4 when the patient is not allowing to touch the area [21].

### 3.4 Foot and Ankle Ability Measure (FAAM)

The Foot and Ankle Ability Measure (FAAM) is a reliable and validated instrument widely recognized for assessing functional ability in patients with foot and ankle conditions. It has two subscales - the Activities of Daily Living (ADL) and the sports scale. The ADL scale assesses ability in performing everyday activities like walking on flat surfaces, going up and down stairs, and putting on footwear, while the sports scale evaluates more demanding tasks such as running, jumping, and cutting. Each activity is rated on a scale from 0 (extreme difficulty) to 4 (no difficulty). The overall FAAM score is calculated as a percentage of the total possible score, providing a quantitative measure of functional impairment and recover [12,22].

### 3.5 Horizontal jump

Lower limb explosive power was assessed using the Standing Broad Jump (SBJ) test. The SBJ test involved having the players stand with both feet on level ground at a marked line, with their arms positioned by their sides. Participants were instructed to jump as far forward as possible, and the distance from the marked line to the point of heel contact was recorded in centimeters. Three jumps were performed, and the best jump was selected for data analysis purposes [23].

### 3.6 Vertical jump

Vertical jump height (VJH) was assessed using a standardized testing protocol [23,24]. Each player stood side-on to a wall, with both feet on the ground, and reached up with one straight hand to touch the wall at the highest point possible with the tip of their middle finger, which was marked with ink. Next, participants were instructed to perform a static squat before jumping as high as possible, marking the wall again with the tip of their middle finger at the highest point reached. Three trials were performed, and the best jump height was used for analysis.

### 3.7 Procedure

**Fig. 3:**
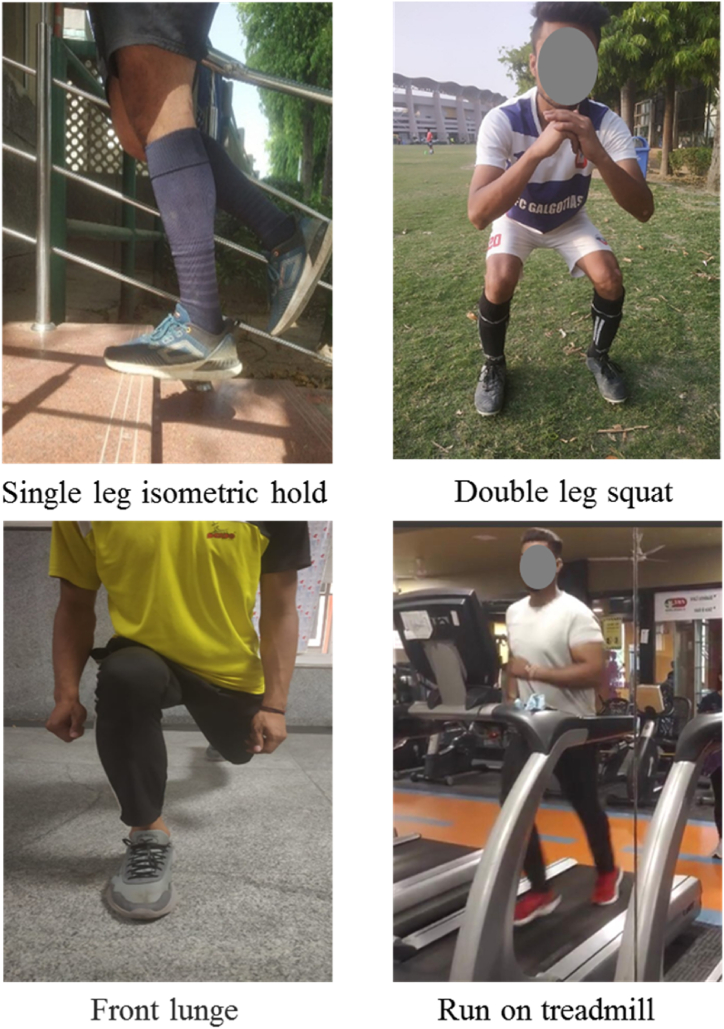
**Rehabilitation and Conditioning Exercises for Ankle Impingement** Top left: Eccentric heel drop exercise performed on a step to strengthen the lower leg muscles and improve ankle stability. Top right: Squat position focusing on maintaining proper alignment to strengthen the quadriceps and glutes without stressing the ankle. Bottom left: Front lunge performed in a gym setting, demonstrating the technique for enhancing hip, knee, and ankle coordination and strength. Bottom right: Treadmill workout emphasizing the controlled foot placement to promote ankle strength and recovery during dynamic movement.

An adapted and comprehensive nonsurgical management program [9,25,26] tailored specifically for posterior ankle pain in football players was implemented over an 8 week period, following a phased approach(Table 1, Fig. 3). Baseline measurements for the study were recorded at day 0, with subsequent assessments conducted after 4 weeks (Phase 2) and 8 weeks (upon completion of Phase 4 training).

**Table 1:** Conservative training for the management of posterior ankle impingement (Fig. 3).

Progression-Orthopedic testing: all negative (except for posterior impingement during hyperplantarflexion) and heel Thrust test. ROM pain free, except active and passive plantarflexion (NPRS score maximum: 6/10)					
**Week**	**Goal**	**Exercise Type**	**Exercises**	**Sets**	**Duration**
**Phase 1** (0–2 weeks)	• Improvement of static ankle stability • Improvement of dynamic ankle stability • Partial ankle plantar flexion was begun (5°)	Closed Kinetic Chain Exercises	Single leg isometric hold	10	5–10 s of hold
			Alternate single and double leg squat/stable surface up to 60 degrees of knee flexion	4	5–15 s of hold
			Balancing over dynadisc with injured ankle with alternate eyes open/eyes closed	3	1 min hold
**Progression-** ROM pain free for all ankle movements except passive plantarflexion (NPRS: maximum 4/10)					
**Phase 2** (3–4 weeks)	Static and Dynamic ankle stability enhancement	Closed kinetic chain exercises majorly focusing on Lunges and squats	Lunges in multiple directions	2 (in front, back & side)	4 reps in each direction
			Bilateral Lunge (eyes open/eyes closed)	2	4
			Bipedal squats on Dynadisc-for dynamic ankle stability		Progression from (2x 10 eyes opened and 1x10 eyes closed) to (3x10 eyes closed)
**Progression-**Pain with passive plantar flexion at end range only (NPRS score maximum, 4/10). Subjective improvement in ankle stability (unipedal stance). Ability to do all exercises without discomfort and pain					
**Phase 3** 5–6 weeks)	• Increasing ankle load tolerance. • Progression to 10° of ankle plantar flexion. • Introduction to plyometrics for improving power and dynamic stabilization of ankle	Closed kinetic chain exercises focusing on dynamic stabilization, lower limb plyometrics	2 legged front jump Landing on one leg	2	1 min
			2 legged jump 90° turn in the air Landing on 1 leg (both sides)	2	1 min
			Front Jump on 1 leg	2	10 reps
			Side jump on 1 leg	2	10 reps
			Rope jumping (Alternate day)	5	1 min
			Walk/run intervals on treadmill (Alternate Day)	10 min	3 days/week
			Side step up and down	3	1 min
			Unipedal stance with other leg Theraband swings (multiple destabilizations with Theraband swings)	4	1 min
			Lunge with isometric abduction (Theraband)	3	8 each leg
**Progression-No pain with CKC/plyometrics and landing exercises**					
**Phase 4** (7–8 weeks)	Return-to-play without pain	Sports specific exercises focusing on running	Running patterns with acceleration/deceleration	2	minimum 10 min
			Cut and turns	2	5 each direction
			Forward and backward running	2	5 each direction
			Rope jumping program continued between running exercises every other day		20 min

### 3.8 Statistical analysis

All analyses were performed using SPSS version 23.0 (IBM Corporation, Armonk, NY, USA) for Windows, and a p value < 0.05 was considered statistically significant. Descriptive statistics summarized the subjects' baseline characteristics, reported as mean and standard deviation. Normality of data was confirmed using the Shapiro–Wilk test. A one-way ANOVA was used to compare the outcome measures at baseline, after 4 weeks and 8 weeks of nonsurgical management for posterior ankle impingement. The Greenhouse–Geisser correction was applied if Mauchly’s test indicated violation of sphericity (p ≤ 0.05). Post hoc analysis with Bonferroni correction was utilized for multiple comparison of all outcome variables. The effect size for each comparison was calculated using Cohen’s d (with thresholds of small = 0.2, medium = 0.5, and large = 0.8 for effect sizes) and partial eta squared (with small = 0.01, medium = 0.06, and large = 0.14 as benchmarks) to evaluate the magnitude and importance of the differences, providing a comprehensive insight into the impact of the interventions [27].

## 4 Results

Fifteen (n = 15) male professional footballers were ultimately included in the study from an initial pool of 85 assessed for eligibility. After excluding 65 players due to various reasons, 20 were enrolled, with five subsequently dropping out due to inability to continue training (Fig. 1). The final participants had a mean age of 25.40 ± 2.95 years (range 19–30 years) and BMI of 19.97 ± 2.47 kg/m^2^. These football players were playing at different positions of which 40 % were midfielder, 35 % forward and 25 % defender. Further, of all the football players 45 % complained of having pain in the dominant foot due to overuse injury while 55 % had pain in the non-dominant foot.

**Fig. 4:**
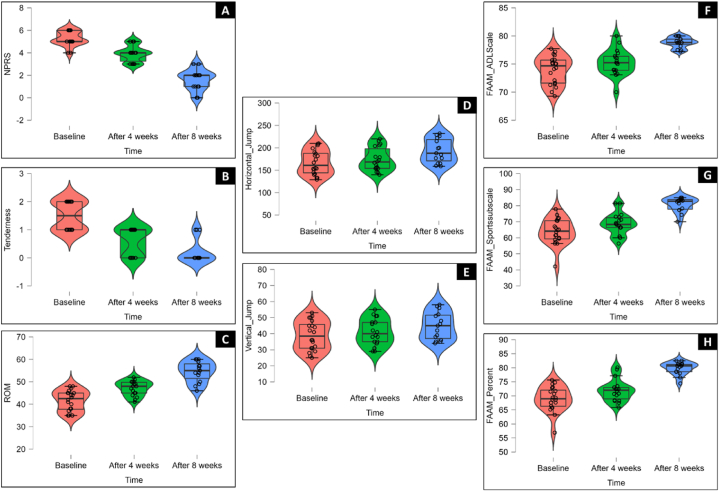
Comparison of the mean of different outcome variables (A)NPRS, (B) tenderness, (C) ROM, (D) horizontal jump, (E) vertical jump, (F) FAAM ADL Scale, (G) FAAM Sports Subscale, (H) FAAM% at baseline, after 4 weeks and after 8 weeks.

Significant changes were observed in various outcome measures over the 8-week intervention (Fig. 4). The results of one-way analysis of variance (ANOVA) showed that the pain levels (Fig. 4A) decreased significantly (p < 0.001), along with reduced tenderness (p < 0.001; Fig. 4B) and improved range of motion (p < 0.001; Fig. 4C). Functional performance measures, including horizontal jump (p = 0.019; Fig. 4D) showed significant improvement in contrast to vertical jump (p = 0.146; Fig. 4E) where the improvement was not significant. Moreover, participants' functional abilities assessed through the Foot and Ankle Ability Measure (FAAM) showed substantial enhancements, with both the ADL subscale (p < 0.001; Fig. 4F) and Sports subscale (p < 0.001; Fig. 4G) displaying significant improvements, leading to an overall higher FAAM% (p < 0.001; Fig. 4H). These findings underscore the potential effectiveness of nonsurgical therapy in ameliorating symptoms and enhancing functionality related to posterior ankle discomfort (Table 2).

**Table 2:** Comparison of outcome variables between group means and the variability within each group using ANOVA (Analysis of Variance).

Outcome Variables	Time	N	Mean	SD	95 % Confidence Interval		Shapiro-Wilk		Mean Square	F	p	η^2^p
					Lower	Upper	W	p				
NPRS	Baseline	20	5.25	0.64	4.95	5.55	0.88	0.06	57.49	101.6	<0.001	0.80
	After 4 weeks	18	3.94	0.73	3.58	4.31	0.82	0.07				
	After 8 weeks	15	1.60	0.91	1.10	2.10	0.88	0.05				
Tenderness	Baseline	20	1.50	0.51	1.26	1.74	0.79	0.15	7.897	33.8	<0.001	0.575
	After 4 weeks	18	0.61	0.50	0.36	0.86	0.85	0.18				
	After 8 weeks	15	0.20	0.41	−0.03	0.43	0.84	0.10				
ROM	Baseline	20	41.55	4.61	39.39	43.71	0.91	0.06	722.4	41.4	<0.001	0.623
	After 4 weeks	18	47.17	3.22	45.56	48.77	0.95	0.38				
	After 8 weeks	15	54.53	4.58	52.00	57.07	0.93	0.25				
Horizontal Jump	Baseline	20	167.15	27.09	154.47	179.83	0.93	0.13	3024	4.29	0.019	0.147
	After 4 weeks	18	174.22	26.07	161.26	187.19	0.90	0.07				
	After 8 weeks	15	193.27	26.36	178.67	207.87	0.91	0.14				
Vertical Jump	Baseline	20	38.90	9.09	34.65	43.15	0.94	0.20	149.8	2.00	0.146	0.074
	After 4 weeks	18	41.11	8.21	37.03	45.19	0.95	0.41				
	After 8 weeks	15	44.80	8.55	40.06	49.54	0.91	0.16				
FAAM ADL Subscale	Baseline	20	73.93	2.52	−0.33	2.98	0.94	0.29	106.13	23.90	<0.001	0.49
	After 4 weeks	18	75.26	2.30	3.15	6.63	0.97	0.87				
	After 8 weeks	15	78.82	0.91	−5.35	−1.79	0.91	0.13				
FAAM Sports Subscale	Baseline	20	63.92	8.09	−0.64	10.01	0.95	0.41	1259.7	27.3	<0.001	0.522
	After 4 weeks	18	68.61	6.79	11.28	22.48	0.96	0.53				
	After 8 weeks	15	80.80	4.43	−17.92	−6.46	0.84	0.05				
FAAM%	Baseline	20	68.93	4.47	0.05	5.96	0.95	0.39	524.40	37.10	<0.001	0.60
	After 4 weeks	18	71.94	3.89	7.78	13.99	0.93	0.20				
	After 8 weeks	15	79.82	2.24	−11.06	−4.71	0.91	0.12				

The post hoc multiple comparison analysis, with Bonferroni correction, revealed significant differences in mean values at three time points: baseline, after 4 weeks, and after 8 weeks (Table 3). Significant changes were seen in mean NPRS and ROM values when comparing baseline to measurements after 4 weeks, baseline to measurements after 8 weeks, and measurements after 4 weeks to those after 8 weeks. Tenderness showed significant differences following nonsurgical management between baseline and measurements after 4 weeks and 8 weeks. However, there were no significant differences in tenderness between measurements after 4 weeks and those after 8 weeks.

**Table 3:** A post-hoc comparison of outcome variables at baseline, after 4 weeks, and after 8 weeks.

Outcome Variables	Time	Time	Mean Difference	SE	t	p	Cohen's d	95 % Confidence Interval	
								Lower	Upper
NPRS	Baseline	After 4 weeks	−1.31	0.244	−5.34	<0.001	−1.74	−2.48	−0.996
	Baseline	After 8 weeks	−3.65	0.257	−14.21	<0.001	−4.85	−6.04	−3.66
	After 4 weeks	After 8 weeks	2.34	0.263	8.91	<0.001	3.12	2.18	4.06
Tenderness	Baseline	After 4 weeks	0.889	0.157	5.66	<0.001	1.839	1.089	2.59
	Baseline	After 8 weeks	1.3	0.165	7.88	<0.001	2.69	1.817	3.56
	After 4 weeks	After 8 weeks	0.411	0.169	2.43	0.056	0.851	0.128	1.57
ROM	Baseline	After 4 weeks	−5.62	1.36	−4.14	<0.001	−1.34	−2.05	−0.638
	Baseline	After 8 weeks	−12.98	1.43	−9.1	<0.001	−3.11	−4.03	−2.179
	After 4 weeks	After 8 weeks	−7.37	1.46	−5.04	<0.001	−1.76	−2.55	−0.976
Horizontal Jump	Baseline	After 4 weeks	−7.07	8.62	−0.82	1.00	−0.266	−0.921	0.388
	Baseline	After 8 weeks	−26.12	9.07	−2.881	0.017	−0.984	−1.698	−0.27
	After 4 weeks	After 8 weeks	−19.04	9.28	−2.052	0.136	−0.717	−1.434	−6.40e−4
Vertical Jump	Baseline	After 4 weeks	−2.21	2.81	−0.787	1.00	−0.256	−0.91	0.3989
	Baseline	After 8 weeks	−5.9	2.95	−1.997	0.154	−0.682	−1.382	0.0174
	After 4 weeks	After 8 weeks	−3.69	3.02	−1.22	0.685	−0.427	−1.134	0.2809
FAAM ADL Subscale	Baseline	After 4 weeks	1.33	0.685	1.94	0.176	0.629	−0.0357	1.294
	Baseline	After 8 weeks	4.89	0.72	6.79	<0.001	2.32	1.4906	3.149
	After 4 weeks	After 8 weeks	−3.57	0.737	−4.84	<0.001	−1.691	−2.471	−0.911
FAAM Sports Subscale	Baseline	After 4 weeks	4.69	2.2	2.13	0.116	0.69	0.0233	1.36
	Baseline	After 8 weeks	16.88	2.32	7.28	<0.001	2.487	1.6385	3.34
	After 4 weeks	After 8 weeks	−12.19	2.37	−5.14	<0.001	−1.797	−2.5862	−1.01
FAAM%	Baseline	After 4 weeks	3.01	1.22	2.46	0.052	0.80	0.13	1.47
	Baseline	After 8 weeks	10.89	1.28	8.48	<0.001	2.90	2.00	3.79
	After 4 weeks	After 8 weeks	−7.88	1.31	−5.99	<0.001	−2.10	−2.91	−1.28

p < 0.05 was significant for study.

Regarding horizontal jump performance, there was no significant change between baseline and measurements after 4 weeks. However, with continued nonsurgical management, there was a significant improvement when comparing baseline measurements to those after 4 weeks. There were no significant differences in mean tenderness values between measurements after 4 weeks and those after 8 weeks.

Nonsurgical management of posterior ankle impingement did not result in significant changes in vertical jump height when comparing mean values at baseline to those after 4 weeks, baseline to those after 8 weeks, or measurements after 4 weeks to those after 8 weeks.

Within the FAAM assessment, consisting of ADL and sports sub-scales, no significant differences were noticed when comparing baseline measurements to those after 4 weeks. However, with the progression of nonsurgical management to 8 weeks, significant changes were observed between baseline measurements and those after 8 weeks, as well as between measurements after 4 weeks and those after 8 weeks. This suggests a positive impact on functional abilities as the intervention advanced.

## 5 Discussion

Posterior ankle impingement (PAI) affects athletes, dancers, and others in activities involving repetitive ankle plantar flexion and recurrent lateral ligament ankle sprains [5]. Nonsurgical management including corticosteroid injections is a standard treatment for PAI. Yet, the effectiveness of nonsurgical management without corticosteroids lacks extensive research [9]. Our study aims to address this gap by evaluating the impact of nonsurgical therapy, without corticosteroids, on the functional outcomes of football players with PAI. While there is limited consensus on the most effective treatment approach for PAI in football players, the existing literature suggests that nonsurgical management is effective in managing the symptoms of PAI.

The prevalence of PAI syndrome varies across different sports and is particularly notable in activities that involve extreme plantarflexion. In the present study, the prevalence was found to be 23.5 %. In professional ballet dancers, PAI syndrome is prevalent, with studies indicating that up to 60 % may experience symptoms during their careers [25]. Another study found that 31.9 % of professional ballet dancers' ankles exhibited posterior impingement, highlighting a significant occurrence of PAI syndrome in this group [28]. Additionally, a study involving patients with ankle impingement identified that 35.8 % had posterior impingement, though it did not provide a broader population prevalence [29]. These findings underscore the significant impact of PAI syndrome in populations engaged in repetitive and strenuous ankle movements. The limited research on prevalence and management of PAI syndrome might be due to diagnostic challenges as its symptoms closely mimic those of conditions like Achilles tendinopathy or retrocalcaneal bursitis.

The results of the study demonstrated that the nonsurgical physiotherapy treatment, particularly progressive loading exercise program, significantly improved symptoms associated with PAI in footballers. This finding is consistent with other studies reporting high rates of symptom resolution and functional improvement through similar nonsurgical management approaches [5,15]. Nonsurgical management for PAI has shown effectiveness with one systematic review reporting 81 % effectiveness rate, although these studies often included corticosteroid injections complicating the assessment of nonsurgical measures alone [30]. Another study found that 60 % of ankles responded to nonsurgical treatments, including ice, rest, and anti-inflammatories among athletes [31].

Further, the present study aligns with these finding, demonstrating significant reductions in symptoms associated with PAI after four and eight weeks of nonsurgical management in football players. These results are consistent with a similar outcomes reported in other study on athletic populations [25].

This study utilized the Foot and Ankle Ability Measure (FAAM) to evaluate functional changes in patients with PAI. Significant increases in both FAAM ADL and Sports subscale were observed after 8 weeks of nonsurgical management with no changes noted at the 4 weeks mark compared to baseline. Related research confirms the FAAM’s sensitivity in detecting rapid functional improvements post-injury and its effectiveness in measuring progress after rehabilitation programs in patients with varied ankle conditions [22,32,33].

Vertical jump is crucial in football for actions such as catching passes and blocking kicks. In the present study, nonsurgical management did not improve vertical jump height in football players with PAI after 4 weeks or 8 weeks, aligning with findings that similar program including plyometrics, weightlifting, and sprinting had no significant effect on collegiate football player’s vertical jump performance [34]. Conversely, horizontal jump improved significantly following 8 weeks and not after 4 weeks of nonsurgical management. Research indicates that chronic ankle instability (CAI) adversely affects jump-landing performance, likely due to compromised neuromuscular control and proprioception [35]. Similarly individuals with ankle sprains exhibit reduced jump performance potentially due to altered muscle activation and limited range of motion [36].

## 6 Limitations

The study on the management of PAI has some limitations. Muscle strength, an important factor in PAI management, was not assessed. Other factors like tibial torsion and Q angle, which influence lower limb biomechanics, were not considered limiting the generalizability of the study findings. The study was limited to male footballers, and caution should be taken when applying the findings to other populations. The small sample size further limits the generalizability and statistical power of the findings. The study did not assess the improvement in proprioception, which can be affected by PAI. The changes in the radiographs was not seen in the study. Additionally, no follow-up assessments were taken to determine the long-term effects of nonsurgical management. Lastly, the absence of a comparison group undergoing standard of care treatment is a noteworthy limitation in this study. The inclusion of such a group would have allowed for a more robust assessment of the effectiveness of nonsurgical management by enabling direct comparisons with the outcomes of standard treatment approaches, thereby providing a more comprehensive evaluation of the intervention's efficacy. Further studies are needed to address these limitations and provide a more comprehensive understanding of PAI management.

## 7 Conclusion

This study concluded that the nonsurgical management-training program without the use of corticosteroids was highly effective in treating posterior ankle pain in football players. The eight-week training period led to significant improvements in pain, range of motion, horizontal jump height, and overall quality of life as measured by the FAAM score. Although the program had no significant impact on vertical jump height, the overall results of the study indicate that investing in evidence-based treatments and taking a holistic approach to athlete care can lead to better outcomes. Given the lack of a well-established standard of care, our study's findings can help inform future research and treatment guidelines in the field.

## CRediT authorship contribution statement

**Jyoti Gupta:** Writing – original draft, Resources, Methodology, Investigation, Data curation, Conceptualization. **Moattar Raza Rizvi:** Writing – review & editing, Visualization, Validation, Supervision, Software, Formal analysis, Conceptualization. **Ankita Sharma:** Writing – review & editing, Supervision, Resources, Project administration, Methodology, Investigation, Data curation, Conceptualization. **Waqas Sami:** Writing – review & editing, Visualization, Validation, Supervision, Software, Methodology, Formal analysis, Data curation, Conceptualization. **Noof Fahad A Al-Kuwari:** Writing – original draft, Supervision, Resources, Methodology, Investigation, Data curation, Conceptualization. **Fatma Hegazy:** Writing – review & editing, Supervision, Methodology, Investigation, Conceptualization. **Shahnaz Hasan:** Writing – review & editing, Supervision, Resources, Project administration, Methodology, Data curation, Conceptualization.

## Ethical approval

Ethical approval was obtained from the Ethical Committee at the Faculty of Allied Health Science, Manav Rachna International Institute of Research & Studies in accordance to Ethical principles for Medical research involving Human (WMA declaration of Helsinki) having Reference No.: MRIIRS/FAHS/PT/2022-23/S-07 dated 17th February 2022.

## Data availability statement

All data have been included in the article, supplementary materials, or are referenced within the article.

The data supporting the findings of this study are not publicly available due to confidentiality restrictions. However, the data can be made available upon reasonable request, subject to approval and confidentiality agreements.

## Funding statement

The publication of this article was funded by College of Nursing, Qatar University.

## Declaration of competing interest

The authors declare that they have no known competing financial interests or personal relationships that could have appeared to influence the work reported in this paper.

## Abbreviations

PAI: Posterior Ankle Impingement
FAAM: Foot and Ankle Ability Measure
ADL: Activities of Daily Living
NPRS: Numeric Pain Rating Scale
ROM: Range of Motion
MRI: Magnetic Resonance Imaging
CT: Computed Tomography
FHL: Flexor Hallucis Longus
PTFL: Posterior Talofibular Ligament
NSAIDs: Nonsteroidal Anti-Inflammatory Drugs
SBJ: Standing Broad Jump
VJH: Vertical Jump Height
ANOVA: Analysis of Variance
SPSS: Statistical Package for the Social Sciences
BMI: Body Mass Index
ICU: Intensive Care Unit
FIFA: Fédération Internationale de Football Association
REST: Rest, Ice, Compression, and Elevation
CAI: Chronic Ankle Instability
